# Synergy between the alteration in the N-terminal region of butyrylcholinesterase K variant and apolipoprotein E4 in late-onset Alzheimer’s disease

**DOI:** 10.1038/s41598-019-41578-3

**Published:** 2019-03-26

**Authors:** Jacek Jasiecki, Anna Limon-Sztencel, Monika Żuk, Magdalena Chmara, Dominik Cysewski, Janusz Limon, Bartosz Wasąg

**Affiliations:** 1 Faculty of Pharmacy with Subfaculty of Laboratory Medicine, Medical University of Gdańsk, Gdańsk, Poland; 2 Consultant Psychiatry, St. Adalbert Hospital, Copernicus Gdańsk, Gdańsk, Poland; 3 Department of Biology and Medical Genetics, Medical University of Gdańsk, Gdańsk, Poland; 4 Laboratory of Clinical Genetics, University Clinical Centre, Gdańsk, Poland; 5 Mass Spectrometry Laboratory Institute of Biochemistry and Biophysics, Polish Academy of Sciences, Warsaw, Poland; 6 Polish Academy of Sciences, Gdańsk Branch, Gdańsk, Poland

## Abstract

While the life expectancy of the population has increased, Alzheimer’s disease (AD) has emerged as one of the greatest health problems of old age. AD is characterized by neuronal loss and cognitive decline. In the AD brain, there is a decrease in levels of acetylcholinesterase (AChE) and an increase in the levels of the related enzyme butyrylcholinesterase (BChE), that accumulate in plaques and tangles. Apolipoprotein E (ApoE) is a major cholesterol carrier and plays an important role in maintaining lipid homeostasis. *APOE*-ε4 constitutes the most important known genetic risk factor for late-onset AD. It has been proposed that the *BCHE-K* allele (Ala539Thr) acts in synergy with the *APOE*-ε4 allele to promote risk for AD. However, there is insufficient evidence to support a correlation. Most studies focused only on the coding regions of the genes. In this study, we analyzed sequence regions beyond the *BCHE* coding sequence. We found synergy between *APOE*-ε4 and SNPs localized in 5′UTR (rs1126680) and in intron 2 (rs55781031) of the *BCHE-K* allele (rs1803274) in 18% of patients with late-onset AD (n = 55). The results show that the coexistence of the *APOE*-ε4 allele and 3 SNPs in the *BCHE* gene is associated with a highly elevated risk of late-onset AD. SNP (rs1126680) in 5′UTR of the *BCHE* gene is located 32 nucleotides upstream of the 28 amino acid signal peptide. Mass spectrometry analysis of the BChE protein produced by SNP (rs1126680) showed that the mutation caused an in frame N-terminal extension of 41 amino acids of the BChE signal peptide. The resultant variant with a 69 amino acid signal peptide, designated N-BChE, may play a role in development of AD.

## Introduction

Alzheimer’s disease (AD), a neurodegenerative disease associated with cognitive decline is the most common form of dementia in elderly individuals. Approximately 13% of people over the age of 65 and 45% over the age of 85 are estimated to have AD. An estimated 50 million people worldwide lived with dementia in 2017. This number will almost double every 20 years, reaching 131.5 million in 2050^1^. There are several types of human dementia of which AD is the most common^2^. Differentiation between AD and dementia with Lewy bodies, vascular dementia (VaD) and frontotemporal dementia (FTD) can be challenging at present and ambiguous. Diagnosis of AD requires a clinical syndrome of dementia, confirmed by postmortem brain autopsy by detecting cerebral pathology including β-amyloid (Aβ) plaques and tau neurofibrillary tangles (NFTs)^3,4^. However, the presence of Aβ in cognitively normal older individuals indicates that other markers are desirable to facilitate more accurate diagnosis of AD^5–10^.

Alzheimer’s disease (AD) is divided into 2 subtypes: early-onset AD occurring at age 30 to 65 years, with approximately 1% to 6% of all cases and late-onset AD, which is the most common form of AD at age later than 65. The cause for most Alzheimer’s cases is still unknown except for a few percent of cases where mutations in genes *APP*, *PSEN1*, and *PSEN2* are associated with early-onset AD. Multiple genetic and environmental risk factors are involved in late-onset AD pathogenesis^11,12^. Many studies have shown that an imbalance between the production and clearance of amyloid-β (Aβ) forming amyloid plaques, is probably a major contributor to neurodegeneration and disease development. The toxicity of Aβ seems to depend on the presence of microtubule-associated hyperphosphorylated forms of the protein tau, which aggregate and deposit in AD brains as neurofibrillary tangles^13^. On the other hand, other factors can also be involved in AD development.

The results from genome-wide association studies (GWAS) have shown that the presence of the ε4 allele of APOE is the strongest genetic risk factor for AD^14–16^. Apolipoprotein E (ApoE) is a protein involved in lipid transport between cells and tissues and it seems to play a role in Aβ aggregation and clearance. The human *APOE* gene exists in three allelic forms (*APOE*-ε2, *APOE*-ε3, and *APOE*-ε4) which differ only by two amino acids in positions 112 and 158, encoding either cysteine or arginine: ApoE2 (Cys112, Cys158), ApoE3 (Cys112, Arg158), and ApoE4 (Arg112, Arg158). Individuals carrying the *APOE*-ε4 allele are at increased risk of AD compared with those carrying the more common ε3 or/and ε2 alleles. It has been hypothesized that ApoE2 and ApoE3 may enhance the clearance of Aβ more efficiently, compared to ApoE4, and/or ApoE4 promotes Aβ fibrillization more effectively than ApoE2 and ApoE3^17–20^. Previous studies showed genes expression changes associated with lipid metabolism as well as inflammation and a role of ApoE4 in these processes among AD patients^21,22^. Other mechanisms, such as tau phosphorylation, neuroinflammation and a role of BCHE protein in the formation of plaques have been also considered as risk factors for AD. Accumulation of amyloid plaques, is thought to initiate a pathogenic cascade that leads to synaptic dysfunction and neurodegeneration^10^.

Butyrylcholinesterase (Uniprot P06276), also known as plasma cholinesterase or pseudocholinesterase, is a serine hydrolase present in most tissues with the highest levels in plasma and liver^23,24^. BChE has a widespread distribution in the human body and it serves as an inherent protector from damages caused by toxic compounds before they reach acetylcholinesterase (AChE) in synapses. In the brain BChE is found in glia and white matter, and it has been shown to be involved, along with AChE, in cholinergic neurotransmission^25,26^. In the human brain, BChE is mainly expressed in glial cells, particularly astrocytes in contrast to AChE which is found in neurons. Nevertheless, BChE is also found in specific populations of neurons, particularly localized in the amygdala and hippocampus^27^ and thalamus^25,28^. BChE was found in amyloid plaques and neurofibrillary tangles (NFTs), which suggests that the protein may be involved in pathogenesis of AD^29–31^. Other researchers demonstrated that BChE may participate in the transformation of beta-amyloid (Aβ) from an initially benign to an eventually malignant form^32^. The accumulation of BChE in cortical grey matter in association with AD pathology, an area that normally has scant BChE activity, suggests an opportunity to detect this pathology during life by imaging BChE^33,34^. Other findings showed that the most frequent genetic variant of the *BCHE* gene – the K-variant (c.1699G> A, p.Ala539Thr, rs1803274), was considerably less effective in attenuating the accumulation of Aβ fibrils than BChE wild type^35,36^. The association between the BChE-K variant and AD risk in patients carrying the ApoE4 allele has been debated and studied many times, but no definitive correlation has been established, as some support the idea^37–39^, while other researchers remain doubtful^40–42^. Despite the fact that many of these studies are based on meta-analysis, GWAS and cohort study, the results are inconsistent and a role for BChE in AD pathology remains unclear^43,44^.

Nearly all previous reports regarding BChE involvement in the development of AD focused only on the K-variant alteration located in the tetramerization domain of BChE.

Our study shows that mutations in the 5′UTR and intron 2 of the *BCHE* gene increase the probability of an association of the BChE-K variant and *APOE*-ε4 allele with late-onset AD.

## Results

Previous studies reported an association between the K variant (BCHE-K) and late-onset AD risk in carriers of *APOE*-ε4. We attempted to replicate this finding in 55 confirmed AD and 18 age matched controls, while expanding the analysis to include noncoding regions of the *BCHE* gene. Genotype and allele frequencies of the BCHE and *APOE*-ε4 variants analyzed are summarized in Table 1. We found a subpopulation of 10 subjects with compound alterations in *BCHE* (rs1126680, rs55781031, rs1803274) and *APOE*-ε4 alleles among 55 people with late-onset AD. The rs1803274 and *APOE*-ε4 alleles were found in 8 out of 45 AD patients and in 6 out of 18 controls. The mutations were mainly heterozygous so that only one allele carried a mutation and the other allele was wild-type.

**Table 1:** Distribution of *APOE*-ε4 and *BCHE* alleles in AD patients and elderly controls (EC).

APOE4 (rs7412-C and rs429358-C)	Signal peptide (rs1126680)	Intron 2 (rs55781031)	K-variant (rs1803274)	AD	control
+(1 hom, 9 htz)	+(1 hom, 9 htz)	+(1 hom, 9 htz)	+(2 hom, 8 htz)	10	^—^
+(2 hom, 7 htz)	^—^	^—^	+(1 hom, 7 htz)	8	^—^
+(9 hom, 27 htz)	^—^	^—^	^—^	37	^—^
+(6 htz)	^—^	^—^	+(6 htz)	^—^	6
+(12 htz)	^—^	^—^	^—^	^—^	12

**Figure 1:**
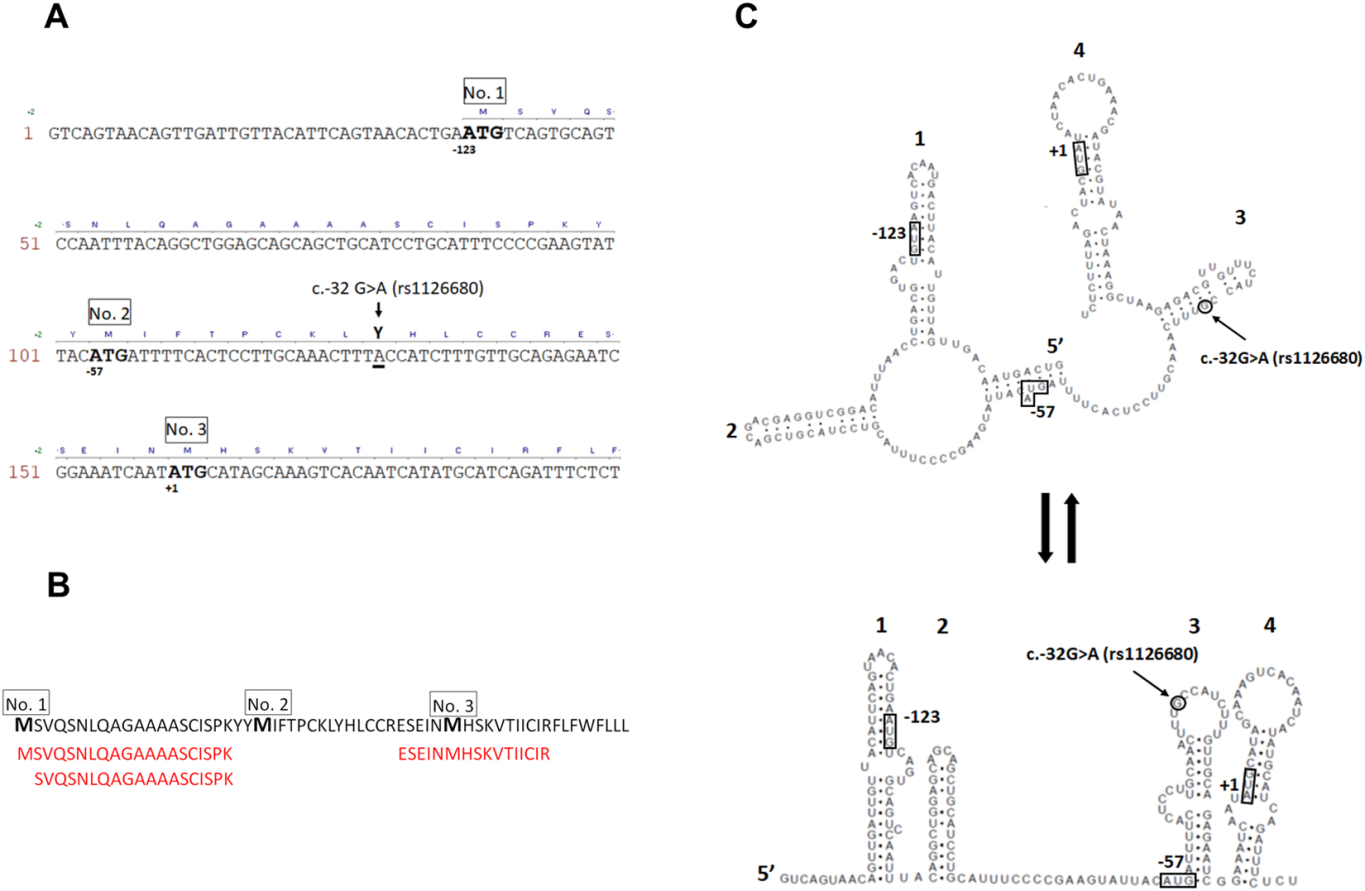
5′ sequence of *BCHE* gene. (**A**) The cDNA and corresponding amino acid sequence of N-terminal part of BChE. Predicted translation initiation codons ATG in the first 200 nt of the *BCHE* cDNA are shown as enlarged bold letters. The proposed new ATG start codon of the extended BChE protein variant is designated No. 1. The known initiation codon of the wt BChE is No. 3. The substitution at nucleotide −32 TGC > TAC (c.−32G >A) p.C-11Y (rs1126680) is enlarged and underlined. (**B**) The peptides of the N-BChE identified by mass spectrometry are shown in bold red under the corresponding sequence of the first 60 aa. (**C**) Influence of alteration c.−32G >A (rs1126680) on two putative secondary structures of the first 200 nt of the *BCHE* mRNA. Two models with different energy values were generated for 37 °C using the MFOLD algorithm^46^ and visualized by RnaViz 2^57^. It is hypothesized that c.−32G> A (rs1126680) substitution (shown in black circle) does not influence the structure of 5′UTR, but probably lowers the affinity of binding proteins. This allows a hairpin to form (comprising Kozak sequence as well as initiation codon AUG No. 3) resulting in changing the initiation codon from No. 3 to No. 1, accompanied by a decrease in protein synthesis.

All 55 late-onset AD patients had *APOE*-ε4. Ten out of 55 late-onset AD patients had 3 *BCHE* alterations (rs1126680, rs55781031, *rs1803274*) and *APOE*-ε4. An additional 8 AD patients had the K-variant mutation and *APOE*-ε4. All 18 elderly controls had *APOE*-ε4. None of the controls had 3 BChE mutations, though 6 out of 12 controls had the K-variant mutation and *APOE*-ε4. SNP rs1126680 is located 32 nucleotides upstream from the ATG start site at codon −28 (initiation codon of the wt BChE- No. 3 in Fig. 1), earlier described as the −116A variant. Full table in Supplementary Table S1. The letter C in APOE4 rs429358-C represents mutation of TGC (Cys) to CGC (Arg) at residue 112 (130 including the signal peptide) in UniProt accession P02649, and the presence of CGC (Arg) at residue 158 (176) in rs7412. Hom stands for homozygous, htz stands for heterozygous.

Alterations in noncoding regions of a gene can play various roles in protein synthesis. 5′ UTR modifications can change the protein translation start site and modifications in introns can be involved in splicing regulation and gene expression pattern. To determine whether rs1126680 and rs55781031 (c.−32G> A and c.1518-121 T > C) affected the protein sequence of serum BChE, we isolated BChE from homozygous individuals from our serum collection^45^ by affinity chromatography on Hupresin and size exclusion chromatography. Isolated proteins were analyzed by mass spectrometry of trypsin-digested proteins. Peptides were found that corresponded to a new translational start site of BChE (Fig. 1A,B, Supplementary Fig. S2) located −69 amino acids from Glu1 of the mature, secreted BChE protein. Furthermore, the extended 69-residue signal peptide was not clipped off the secreted BChE protein. To predict the rs1126680 effect on local RNA secondary structure of the first 200 nucleotides of the BChE transcript MFOLD algorithm and RNAsnp Web Server were used^46–48^. We did not observe significant changes in the predicted secondary structures outside of the region containing the alteration (Fig. 1C).

Sequence analysis using the Kozak sequence prediction algorithm showed that the new translation start site at residue −69 (nucleotide −123 in Table 2) scored higher for the Kozak consensus sequence than the start site at residue −28 in wild-type BChE (nucleotide + 1 in Table 2). A perfect Kozak sequence would have a score of 1. Scores of 0.17 and 0.14 for the 2 ATG start sites in Table 2 indicate that BChE expression levels are modulated through inefficient start site sequences. The slightly higher score of 0.17 suggests the homozygous mutant would have higher plasma BChE activity. Contrary to expectation, plasma samples with 3 BCHE SNPs had a lower BChE activity. The N-terminus extended BChE protein variant, designated N-BChE, is 41 aa longer than wt and carries a substitution at nucleotide −32 position TGC > TAC (c.−32G > A) p.C-11Y (rs1126680) (Fig. 1).

**Table 2:** The ATGpr computer program was used to predict the initiation codons of the *BCHE* gene^58^.

No. of ATG from 5′ end	Position of protein start*	Reliability	Identity to Kozak rule A/GXXATGG	Sequence (first 7 aa)	ORF Lenght (aa)
1	−123	0.17	tXXATGt	MSVQSNL	643
2	−57	0.06	tXXATGa	MIFTPCK	621
3	+1	0.14	AXXATGc	MHSKVTI	602

Two additional putative initiation codons (No. 1 and 2) were found in the same open reading frame as the known one (No. 3) as shown in Fig. 1. The calculated reliability score of 0.17 shows that initiation codon No. 1 has the best match to the Kozak translation initiation site. *-Numbering of positions of the first nucleotide-A in ATG of the predicted translation initiation codons in the transcript is described according to position of the first nucleotide-A in ATG of the known one (No. 3).

## Discussion

Genetically, the ε4 allele of the apolipoprotein E (*APOE*) gene is the strongest risk factor for late-onset AD^49^. Several competing hypotheses explain the cause of the disease. An early hypothesis, that AD is defined by the presence of Aβ plaques and neurofibrillary tangles became inadequate when it was found that the brains of up to 40% of cognitively normal older adults also had Aβ plaques and tangles. Additional markers of AD are required to facilitate a more accurate diagnosis of the disease. This study shows that the coexistence of the *APOE*-ε4 allele and 3 SNPs in the *BCHE* gene, localized in 5′UTR (rs1126680) and in intron 2 (rs55781031) of the *BCHE-K* allele (rs1803274), is associated with a highly elevated risk of late-onset AD.

Little is known about regulation of *BCHE* gene expression. Our previous studies and results presented by others have shown that c.−32G> A (rs1126680) (also known as −116A) substitution in exon 1 was associated with lower BChE activity^45,50^. It was shown that 5′ UTR c.−32G> A, (rs1126680) variant is preferentially found in cis with K variant (c.1699G> A, p.A567T) and intron 2 alteration (c.1518-121 T > C, rs55781031). It was also discussed that c.−32G> A is responsible for lowering of BChE activity by affecting transcription and/or translation. It was considered that 5′ UTR c.−32G> A, (rs1126680) variant can directly modulate protein translation through alterations in mRNA secondary structure. Protein translation is often regulated by mRNA secondary structure. Complementary sequences within the 5′ UTR can form stable stem-loop structures that may disturb initiation of translation^51,52^. A theoretical model of the mRNA secondary structure of the *BCHE* 5′ UTR region presented in Fig. 1C was predicted using mFold^46^. It is possible that the position of substitutions in the loop of the stem could affect binding of the regulatory proteins and disturb translation efficiency. Such a process was previously described for 5′ UTR of the ferritin receptor mRNA, which contains a stem loop that recruits binding proteins and controls translation efficiency^53^. Here, we observed two effects of c.−32G> A, (rs1126680): change in initiation codon of BChE protein resulting in N-extension of the 41 amino acids of the protein and alteration in the extended amino acid sequence p.C-11Y. Furthermore, we showed that the N-extension of the BChE protein was associated with some cases of late-onset AD. It is noteworthy, that N-terminally extended membrane variants of AChE were observed in brain neurons and hematopoietic cells as an effect of alternative splicing^54^. Extended N-AChE proteins may have transmembrane domains at their N terminus and play a role in apoptosis^55^. Our research shows N-terminally extended BChE variant produced as an effect of alternative translation start.

Our results show no association between AD risk in patients carrying the *ApoE4* allele and the BChE K-variant when no other *BCHE* gene mutations are evaluated. This conclusion is based on the finding that 6 out of 18 control cases (33%) and 18/55 AD patients (33%) were positive for the BChE K-variant. However, a strong association (100%) was found between late-onset AD in patients carrying the *ApoE4* allele and the BChE K-variant when 2 additional mutations in noncoding regions of the *BCHE* gene were present.

Different types of dementia may be caused by different molecular mechanisms. Many factors can be involved in development of late-onset AD. In this study, we found synergy between *APOE*-ε4 and SNPs localized in the *BCHE* gene (rs1126680, rs55781031, rs1803274) in 18% of patients with late-onset AD (n = 55). Despite the small number of patients, we propose that the presence of the *APOE*-ε4 allele and 3 SNPs in the *BCHE* provides 100% confidence in predicting late-onset AD. These multiple gene alterations were present only in patients with late-onset AD. The limitation of our study is that only 18% of late-onset AD patients carried all these mutations. A larger cohort study that considers additional genetic and environmental factors may account for the 82% of late-onset AD cases that did not contain this set of mutations.

## Materials and Methods

AD patients (n = 55) enrolled in the study were selected by specialists in geriatric psychiatry as described earlier^56^. Briefly, the diagnosis of AD was based on medical interviews, clinical symptoms, appropriate imaging examinations and clinical tests including the Hachinski Ischemic Scale, the Geriatric Depression Scale, the Mini Mental State Examination (MMSE) and the Clock Drawing Test. In order to exclude other possible causes of impairments in cognitive function, complete blood count, a lipidogram and other tests clinically appropriate for somatic diseases were performed during the diagnostic process. The patient group consisted of 26 males and 29 females with a mean age of 83 years (range 65–102 years). The control group (n = 18) had no signs or symptoms of dementia or a severe somatic disorder. Controls consisted of 9 males and 9 females with a mean age of 76 years (range 68–87 years). All participants were of European origin and homogeneous ethnic (Polish) background. Serum collection and genotyping of the control group (n = 1200) was described earlier^45^.

### DNA Extraction, PCR Amplification and Sanger Sequencing

Genomic DNA was extracted from 1 mL of peripheral blood leukocytes using Blood Mini kit in accordance with the manufacturer’s protocol (A&A Biotechnology, Poland). Sequences of 4 amplicons of the *BCHE* gene were determined using the method previously described^45^. Briefly, 4 regions of the *BCHE* gene were amplified by PCR using the following primers M1F, 5′-F- AGACTACCTGCAATTGTAAAGCA, and M1R, 5′-TCTCATCCCACAGAATGAGC; M2-2F, 5′- GCCACAGTCTCTGACCAAGTG and M2-2R-5′- TTCTGTTCCTAGCTTCATAAAGAG; M3-F, 5′- CACTAAGCCCAGTTCACATACG, M3-R, 5′- CATCACCGTGCCTTGGAG; M4-1F, 5′- TGTACTGTGTAGTTAGAGAAAATGGC; M4-1R, 5′- TACTAAGTTAAAGATGTGAGGAATC.

*APOE*- ε2/ε3/ε4 alleles (rs429358, rs7412) polymorphism were determined using the following primers APO4F 5′-ACGCGGGCACGGCTGTCCAAGGAG and APO4R 5′-CTCGCGGGCCCCGGCCTGGTACAC. A 25-μL PCR mixture contained 60 ng of extracted DNA, 10 pmoles of each forward and reverse primer, dNTPs, buffer, and Marathon *Taq* DNA polymerase (A&A Biotechnology, Poland). Amplification was performed with an initial denaturation at 95 °C for 5 minutes, followed by 35 cycles of denaturation at 95 °C for 30 s, annealing at 55 °C for 30 s, and extension at 72 °C for 30 s with a final extension at 72 °C for 10 minutes. PCR products were purified using Clean Up kit (A&A Biotechnology, Poland). Bidirectional DNA sequencing of PCR amplification products was performed using BigDye Terminator v.3.1 cycle sequencing kit and 3130 Genetic Analyzer according to the manufacturer’s protocol (ThermoFischer Scientific, USA). Sequences were analyzed by Sequencher v.4.10 DNA Software (Gene Codes Corporation, USA) and aligned with *BCHE APOE* reference genomic sequences.

### Purification of BChE from serum by affinity chromatography on Hupresin^®^ and mass spectrometry

Human BChE was purified from 0.1 mL of serum on 50 ul of Hupresin AC Sepharose (Chemforase, Mont-Saint-Aignan, France). Hupresin AC was equilibrated with 100 mM NaCl, 20 mM TrisCl pH 7.4 and serum proteins were loaded on the 50 µl resin in 1.5 ml filter tubes and washed using 100 mM NaCl, 20 mM TrisCl pH 7.4 buffer. BChE complexes were eluted with 50 µl of 0.5 M trimethylammonium chloride (TMA Cl) in 100 mM NaCl, 20 mM TrisCl pH 7.4 buffer. Eluted proteins were dialyzed and concentrated to 20 µl in Amicon Ultra 10 K Centrifugal Filters. Subsequently, eluted proteins were applied to a gel filtration chromatography column, TSK gel 3000 SWXL (300 × 7.8 mm, Tosoh) equilibrated with 10 mM phosphate buffer pH 7.4; 8 g/L NaCl at a flow rate of 1 ml/min at RT. The column was connected to a Merck Hitachi LaChrom HPLC system equipped with an L-7420 UV-Vis detector. Eluted fractions with BChE activity were collected and concentrated to 20 µl in Amicon Ultra 10 K Centrifugal Filters. BChE activity of each fraction was determined spectrophotometrically by modified Ellman’s method using BTC (S-butyrylthiocholine iodide) as a substrate. The assay was performed in 96-well microtiter plates in a final reaction volume of 200 μl of 100 mM PB buffer (pH 7.4) with a final concentration of 0.5 mM DTNB (5,5′-dithiobis(2-nitrobenzoic acid)) and 5 mM BTC. The absorbance was monitored at 412 nm by repeated measurements at 1 min intervals for 10 minutes by a thermostated microplate reader spectrophotometer (Tecan Infinite M200Pro) at 25 °C. 50 μL of 100 mM ammonium bicarbonate buffer was added to the each protein sample, reduced with 5 mM TCEP for 30 min at 60 °C, blocked with 10 mM MMTS for 15 min at RT and digested overnight, shaking with 10 ng/ml trypsin (CAT NO V5280, Promega) at 37 °C. Finally, to stop digestion trifluoroacetic acid was added at a final concentration of 0.1%. The digest was centrifuged at 4 °C, 14 000 g for 30 min, to pellet solids. The particle-free supernatant was analyzed by LC-MS/MS in the Laboratory of Mass Spectrometry (IBB PAS, Warsaw) using a nanoAcquity UPLC system (Waters) coupled to an Orbitrap Elite mass spectrometer (Thermo Fisher Scientific). The mass spectrometer was operated in the data-dependent MS2 mode, and data were acquired in the m/z range of 300–2000. Peptides were separated by a 180 min linear gradient of 95% solution A (0.1% formic acid in water) to 35% solution B (acetonitrile and 0.1% formic acid). The measurement of each sample was preceded by three washing runs to avoid cross-contamination. The final MS washing run was searched for the presence of cross-contamination between samples. Data were searched with the Max-Quant (Version 1.5.7.4) platform search parameters: match between runs (match time window 0.7 min, alignment time 20 min), enzyme: trypsin/p; specific; max missed 2, minimal peptide length 7aa, variable modification: methionine oxidation, N-term acetylation, fixed: cysteine alkylation, main search peptide tolerance 4.5 ppm, protein FDR 0.01. Data were searched against home made protein database including isoforms of BChE.

### Ethical approval and consent to participate

All patients and healthy individuals gave written informed consent for molecular genetic testing. The study was approved by the Independent Bioethics Commission for Research at the Medical University of Gdansk. The experiments were done in accordance with the Helsinki Declaration of 1975.

## Supplementary information

Supplementary Table S1.

Supplementary Figure S2.
