# Supplementary figures and images for: Synergy between the alteration in the N-terminal region of butyrylcholinesterase K variant and apolipoprotein E4 in late-onset Alzheimer’s disease
Source: Sci Rep. 2019 Mar 26;9:5223. doi: 10.1038/s41598-019-41578-3 (PMC6435664; doi:10.1038/s41598-019-41578-3)

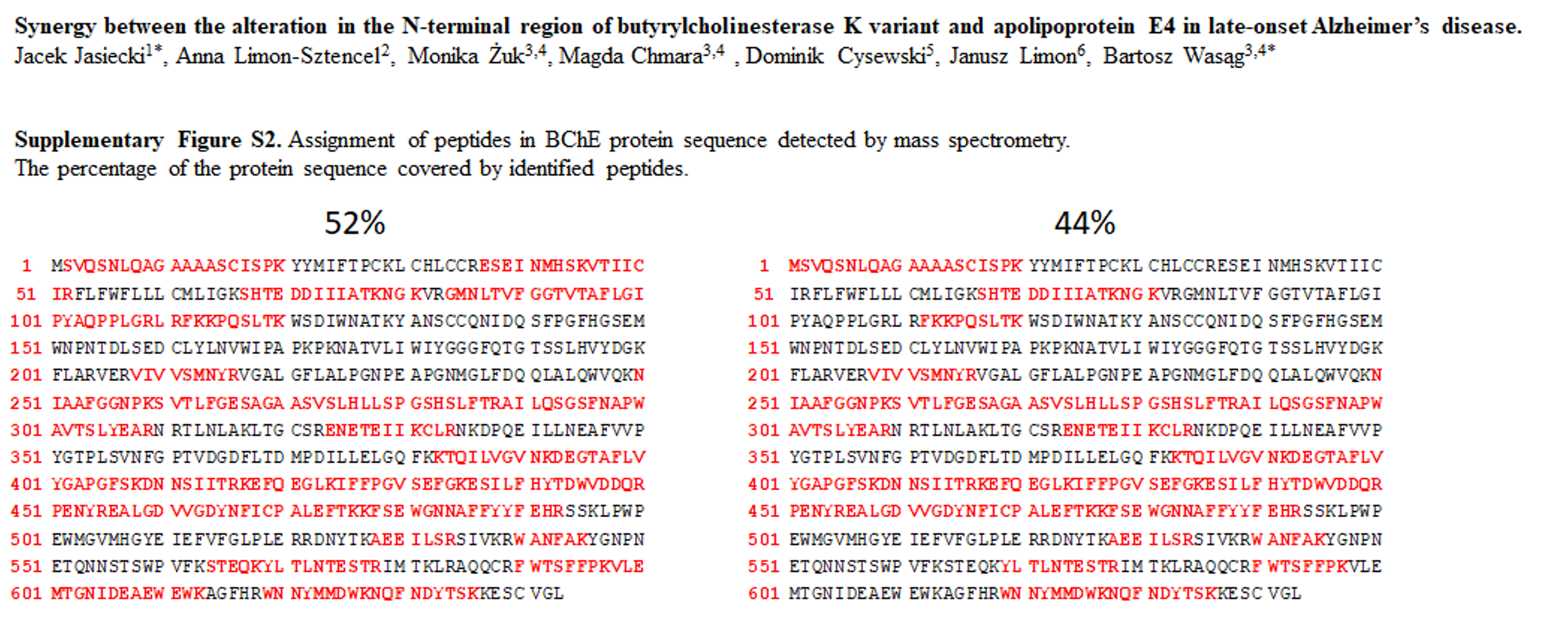

Supplement: Supplementary file 2 — Supplementary Figure S2. [file 41598_2019_41578_MOESM2_ESM.tif]
